# Heart failure with preserved ejection fraction: current status and challenges for the future

**DOI:** 10.1007/s12471-016-0808-8

**Published:** 2016-03-03

**Authors:** J. van der Velden, E. E. van der Wall, W. J. Paulus

**Affiliations:** 1 Department of Physiology, Institute for Cardiovascular Research (ICaR-VU), VU University Medical Center, Amsterdam, The Netherlands; 2 ICIN—Netherlands Heart Institute, Utrecht, The Netherlands; 3 Netherlands Society of Cardiology/Holland Heart House, Utrecht, The Netherlands

Over the past two decades it has become evident that more than 50 % of all heart failure patients suffer of heart failure with preserved left ventricular (LV) ejection fraction (HFpEF), previously called diastolic heart failure because of important involvement of diastolic LV dysfunction. The advances and current status of HFpEF research are described in this special focus issue of the Netherlands Heart Journal. HFpEF is the most common heart failure phenotype in ageing societies, highly prevalent in elderly women and frequently accompanied by comorbidities which result from a detrimental lifestyle, such as obesity, metabolic syndrome, type 2 diabetes mellitus and salt-sensitive arterial hypertension [1]. Despite modern heart failure therapy, the prognosis of HFpEF has not improved over the last decades. By 2020, the prevalence of HFpEF is projected to exceed 8 % of persons older than 65 years of age and because of the current pandemic of obesity, the prevalence of HFpEF in persons younger than 65 years of age is expected to rise exponentially.

A targeted strategy is needed to prevent the progression from obesity and type 2 diabetes towards diastolic LV dysfunction and HFpEF. Available heart failure therapies might exert beneficial effects at an early stage of HFpEF, which is, however, poorly recognised with many patients initially presenting with an advanced form of heart failure. Recognition of an early stage of diastolic dysfunction and initiation of heart failure therapy should be done in primary care, and novel staged therapeutic strategies should be tested to prevent or retard the progression from diastolic LV dysfunction to end-stage heart failure. To battle this life-threatening chronic disease, specialists from different areas of expertise need to join forces.

Based on recent findings in cardiac tissue samples from HFpEF patients, a novel paradigm on HFpEF pathogenesis was formulated [1]. This paradigm proposes that the different comorbidities initiate chronic systemic inflammation, which via perturbations of the microvasculature of the heart stiffens and damages cardiac muscle cells causing diastolic dysfunction. Impaired relaxation not only results from stiffened cardiac muscle cells, but also involves changes of the extracellular matrix and deposition of collagen [2]. The earliest stage of HFpEF should be detected by general practitioners as previously unknown heart failure was found in 27.7 % of patients with type 2 diabetes aged 60 years or over, of which 83 % involved HFpEF. Boonman-de Winter and colleagues [3] emphasise that general practitioners, internists and other healthcare professionals may be unaware of the high prevalence of heart failure in older diabetic patients and describe a disease-management program to detect heart failure in older patients with type 2 diabetes [3]. Also in the clinic, diagnosis of HFpEF remains a challenge with the currently used diagnostic tools. Accurate diagnosis of HFpEF and staging of this complex type of heart failure may require invasive stress testing and accurate assessment of right ventricular function by means of cardiac MRI [4]. Moreover, novel blood biomarkers may become available to better identify and stage HFpEF patients [5, 6]. As HFpEF clinical trials have provided neutral outcomes and because of the magnitude of this type of heart failure, basic scientists and clinicians need to join forces to develop novel treatment strategies to prevent and reverse cardiac dysfunction. Stiffening of the heart muscle may be reversed by targeting protein kinase G and phosphorylation of the giant protein titin [7], which is an important regulator of muscle cell stiffness and relaxation. Newly developed drug therapies should be tested in models which resemble the complex disease phenotype observed in humans [8]. During past years insights have been obtained into the complex pathogenesis of HFpEF, which are summarised in this special issue. The challenge for the future lies in raised awareness of this devastating disease, improved patient stratification and development of stage-specific therapies.
